# Antibiotic Paste as an Intracanal Medicament in Infected Primary Teeth: A Systematic Review

**DOI:** 10.7759/cureus.82876

**Published:** 2025-04-23

**Authors:** Archita Barve, Laxmi Lakade, Preetam Shah, Shweta Chaudhary, Shweta Jajoo, Gandhali Joshi

**Affiliations:** 1 Pediatric Dentistry, Bharati Vidyapeeth (Deemed to be University) Dental College and Hospital, Pune, IND

**Keywords:** antibiotic paste, c. albicans, e. faecalis, intracanal medicaments, primary teeth

## Abstract

Intracanal medicaments, such as antibiotic pastes, in infected primary teeth have been evaluated to treat persistent polymicrobial infections, especially with resistant species like *Enterococcus faecalis* and *Candida albicans*. Advanced formulations such as triple antibiotic paste (TAP), calcium hydroxide (Ca(OH)₂), clindamycin-modified TAP, and nanoparticle-based medicaments have been studied for microbial reduction and clinical outcomes. This systematic review included studies that had evaluated antibiotic pastes for intracanal use in primary teeth. A comprehensive electronic database search was conducted following the Preferred Reporting Items for Systematic Reviews and Meta-Analyses (PRISMA) guidelines, and studies were evaluated for eligibility on a population, exposure, comparator, outcome, and study design (PECOS) framework. A comprehensive search was conducted across seven electronic databases that included PubMed, Scopus, Web of Science, ScienceDirect, Cochrane Library, Embase, and Google Scholar. Risk of bias was evaluated using RoB 2.0 and QUIN tools. Out of 381 studies, 11 studies met the inclusion criteria, which evaluated medicaments such as TAP, Ca(OH)₂, clindamycin-modified TAP, 3C paste, chitosan chlorhexidine (CS-CHX) nanoparticles, and herbal alternatives. Results showed that TAP and its modified forms showed better antimicrobial efficacy, with reductions of up to 99.95% for aerobes and 99.78% for anaerobes. New formulations like nanoparticle-based drugs and 3C paste are promising but need clinical validation. This study suggests the potential of tailored intracanal medicaments in pediatric endodontics.

## Introduction and background

Infected primary teeth that are disinfected and treated endodontically can act as the best space maintainers in the oral cavity till the permanent teeth erupt. The developing permanent dentition is guided by the prior presence of the primary set, which affects mastication and speech development as well as facial aesthetics. If infections or other complications lead to the loss of these teeth before they are replaced by permanent ones, the child might face problems such as malocclusion, speech impairment, and psychological issues [1,2].

The anatomical and physiological characteristics of primary teeth, primarily their relatively thin enamel and dentin, large pulp chambers, and high permeability, make them more susceptible to infections [3]. These infections often have a polymicrobial etiology, but anaerobic and facultative bacteria predominate. Such infections are treated both mechanically and chemically through debridement and disinfection, respectively, to remove pathogens that persist in inaccessible areas of the root canal system [4].

Intracanal medicaments are important in achieving microbial control inside the root canal system of primary teeth [5]. Among all the medicaments available, pastes with antibiotics have garnered much interest due to their wide-spectrum antimicrobial action. One of the most commonly used formulations is TAP, also known as triple antibiotic paste, a combination of ciprofloxacin, metronidazole, and minocycline [6]. Localized application of antibiotics has advantages, including a higher concentration at the site of infection, fewer systemic side effects, and a lower potential for systemic antibiotic resistance. However, the cytotoxic effects and potential tooth discoloration associated with minocycline have moved clinicians to use alternative formulations with comparative studies [7-10].

Other intracanal medicaments, such as calcium hydroxide and chlorhexidine (CHX), have been in use for decades in pediatric endodontics because of their antimicrobial activity and the ability to create an unfavorable environment for bacterial survival [8]. Calcium hydroxide has a high pH and acts through the denaturation of bacterial proteins and neutralization of endotoxins. CHX, on the other hand, shows broad-spectrum antimicrobial activity with substantivity, meaning it remains effective over a considerable period of time [11]. The newer formulations that have come to light include chitosan-based medicaments and nanoparticles, among others; however, there is still ongoing active research into their long-term efficacy and safety [12].

Although promising, antibiotic pastes are not widely practiced, mainly owing to issues surrounding the risk of inducing antibiotic resistance, potential adverse effects, and variability in clinical outcomes described in the literature [10,13]. The localized delivery of antibiotics might also lead to the development of resistance in the microflora of the root canal and oral cavity, which could impede long-term clinical success. To date, the literature presents a diverse range of studies on antibiotic pastes as applied to infected primary teeth; the usage of the latter necessitates the need for a systematic synthesis of this evidence in order to identify therapeutic potential, limitations, and the broader implications of antibiotic pastes in pediatric endodontics.

## Review

Materials and methodology

PECOS Protocol and PRISMA Guidelines

This systematic review was constructed in accordance with the PECOS framework to ensure structured and rigorous inclusion of relevant studies and adhered to Preferred Reporting Items for Systematic Reviews and Meta-Analyses (PRISMA) reporting guidelines [14] to ensure transparency and replicability. The PECOS elements were defined as follows:

P (population): infected primary teeth.

E (exposure): use of antibiotic paste as an intracanal medicament.

C (comparator): other intracanal medicaments such as calcium hydroxide, CHX, chitosan-CHX (CS-CHX) nanoparticles, and propolis.

O (outcomes): antibacterial efficacy and duration of intracanal medicament.

S (study design): randomized controlled trials (RCTs), clinical studies, cross-sectional studies, and in vitro studies.

Inclusion and Exclusion Criteria

The inclusion and exclusion criteria developed for this review are presented in Table 1.

**Table 1:** Inclusion and exclusion criteria developed for this review

Criteria	Inclusion	Exclusion
Population	Infected primary teeth	Permanent teeth and young permanent teeth
Intervention	Antibiotic pastes (e.g., triple antibiotic paste, calcium hydroxide, and chlorhexidine)	Systemic antibiotic treatments
Comparator	Other intracanal medicaments (e.g., calcium hydroxide and propolis)	Studies with no comparator
Outcomes	Antibacterial efficacy and duration of medicament	Outcomes not related to antibacterial efficacy
Study design	RCTs, clinical studies, cross-sectional studies, and in vitro studies	Review articles, irrelevant articles, and case reports
Language	English	Non-English studies
Publication type	Full-text articles	Grey literature and unpublished studies

Database Search Protocol

A comprehensive search was conducted across seven electronic databases that included PubMed, Scopus, Web of Science, ScienceDirect, Cochrane Library, Embase, and Google Scholar. Searches were carried out using Boolean operators and Medical Subject Headings in combination with free-text keywords (Table 2). In addition, filters for study type, language, and date of publication were applied.

**Table 2:** Search strings utilized for the review

Database	Search string
PubMed	("Antibiotic Paste" OR "Triple Antibiotic Paste") AND ("Primary Teeth" OR "Deciduous Teeth")
Scopus	TITLE-ABS-KEY("Antibiotic Paste") AND TITLE-ABS-KEY("Primary Teeth" OR "Infected Teeth")
Web of Science	TS=("Antibiotic Paste" OR "Intracanal Medicament") AND TS=("Deciduous Teeth" AND "Bacterial Pathogens")
ScienceDirect	("Antibiotic Medicaments" AND "Pediatric Dentistry") AND ("Infection Control")
Cochrane Library	MeSH descriptor: [Antibiotics] explode all trees AND MeSH descriptor: [Primary Teeth] explode all trees
Embase	("Antibiotic Intracanal Paste" OR "Triple Antibiotic Paste") AND ("Infected Deciduous Teeth")
Google Scholar	"Antibiotic Paste for Intracanal Use" AND "Primary Teeth Infection"

Data Extraction Protocol

A standardized data extraction form was used by two independent reviewers to extract the data. The items involved included the title of study, authors, year of publication, study design, population characteristics, details of the intervention, comparator details, outcomes assessed, and key findings. Any disagreements were resolved by consulting a third reviewer. The corresponding authors were contacted for clarification on any necessary issues.

Bias Assessment Protocol

In the observational studies, the ROBINS-I instrument [14] assessed confounding bias, selection bias, and measurement bias within seven domains. For RCTs, Cochrane’s RoB 2.0 tool [15] was used to check on random sequence generation, allocation concealment, blinding, incomplete outcome data, and selective reporting. For the in vitro studies, the QUIN tool [16] was utilized. 

Results

**Figure 1:**
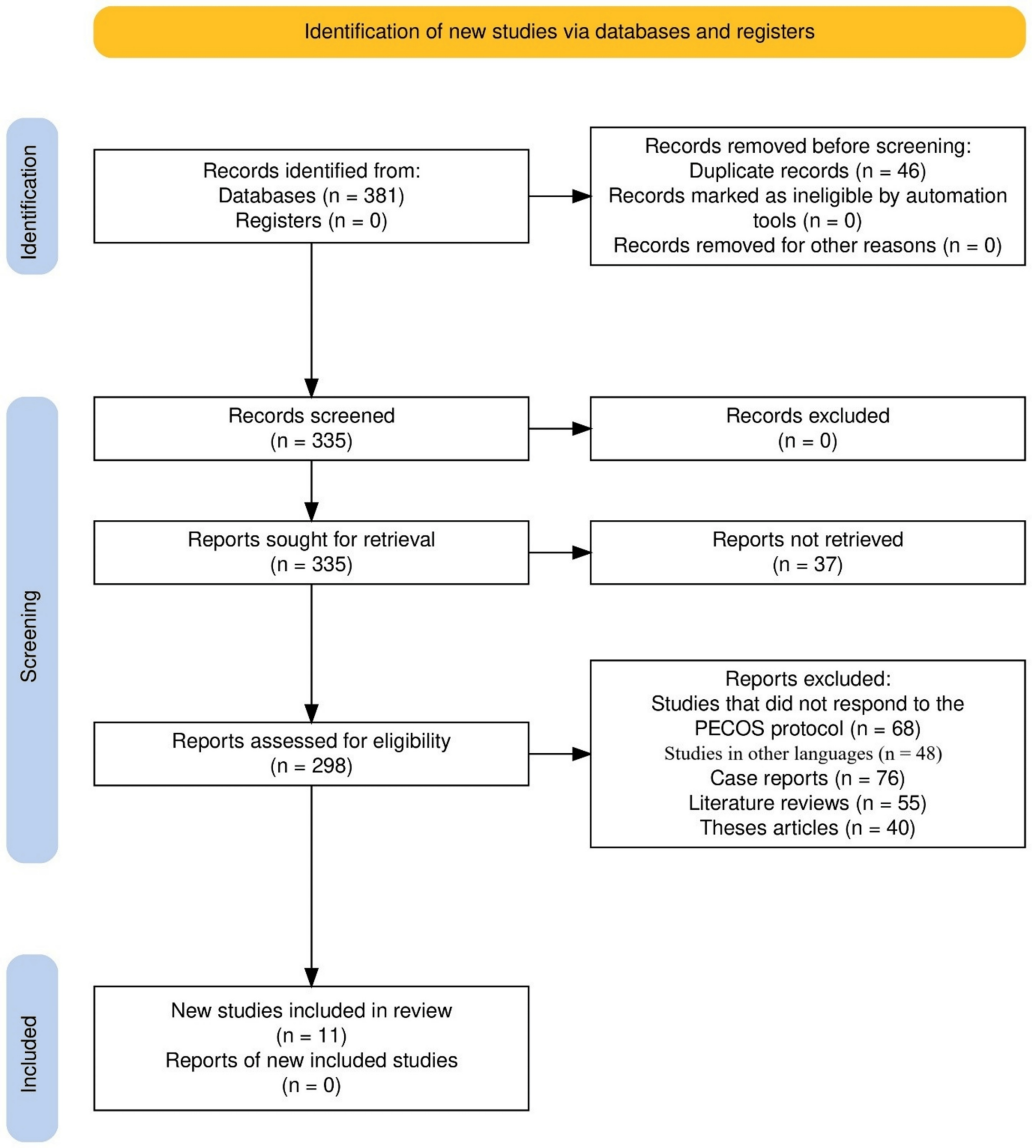
PRISMA flow diagram for systematic review PRISMA, Preferred Reporting Items for Systematic Reviews and Meta-Analyses

Initially, a total of 381 records were identified by database searches, and no records were identified by registers. Forty-six duplicates were removed from the retrieved 335 records; these then advanced into the screening stage. No more records were excluded during this screening process. The reports of 37 were unavailable to retrieve. A total of 298 records went through eligibility assessment, and the studies that did not meet the inclusion criteria included those not using the PECOS protocol (n = 68), conducted in other languages (n = 48), case reports (n = 76), literature reviews (n = 55), and theses (n = 40). Based on the results of these assessments, 11 studies were included in the review, with no other newly identified studies (Figure 1). 

Demographic Characteristics

The studies were geographically diversified (Table 3), conducted in countries like India [17-19], Iran [20,21], Turkey [22], and Egypt. This reflects global interest and variability in clinical and laboratory settings. The publication years are between 2010 and 2024. This implies a changing trend as well as a consistent study regarding intracanal medicaments during the last 10 years [17-27]. The study designs include RCTs, pilot studies, and in vitro experiments, and this balance is represented between the clinically relevant and controlled experimental methodologies. This is all done in an attempt to identify the best intervention [17-27].

**Table 3:** Demographic characteristics of the included studies

Author name	Year	Location	Study design	Sample size	Follow-up period
Ahirwar et al. [17]	2018	India	Randomized control trial	40	3 days
Chandra and Thosar [18]	2024	Wardha, India	In vitro study	Not applicable	Not applicable
Dutta et al. [19]	2017	Bhubaneswar, India	In vivo study	48	7 days
Ghahramani et al. [20]	2020	Iran	Randomized control trial	39	1 week
Gholami et al. [21]	2023	Shiraz, Iran	Randomized control trial	39	7 days
Kargül et al. [22]	2010	Turkey	Pilot study	57	1-4 years
Paikkatt et al. [23]	2018	India	Randomized control trial	34	Not applicable
Qamar et al. [24]	2023	Hazaribagh, India	Randomized control trial	60	7 days
Reddy et al. [25]	2017	India	In vivo study	55	1 year
Verma et al. [26]	2022	Gurugram, India	Randomized control trial	60	12 months
Wassel et al. [27]	2023	Egypt	In vitro study	63	3 and 7 days

The least number of patients was 34 [23]. The highest was 63 for in vitro. This period after the treatment varied largely, which can be as short as three days in the studies of herbs [17] to as long as 12 months in investigations into modified TAPs [26] for both early and late effects of treatment to be assessed.

Clinical and Radiographic Findings

Within clinical results, the microbial inhibition was observed as being consistent between all treatments with TAP, and its modified versions were sometimes significantly more active than comparatives. The combination of TAP with CHX was also highly effective, with enhanced killing capacity of *Enterococcus faecalis* against standalone TAP or calcium hydroxide [20,23]. The highest antibacterial activity was observed with nano-formulations such as CS-CHX nanoparticles, and residual effects were reported for a longer duration even after medicament removal [27].

Significant bacterial reduction was observed for novel 3C paste and herbal alternatives, like *Ocimum sanctum*, compared to TAP formulations, in particular against more resistant organisms like *E. faecalis* [17]. One documented study reported as much as a 99.95% reduction of aerobes and a 99.78% reduction of anaerobes with TAP, pointing out strong antimicrobial activity [21]. Radiographic outcomes were rarely used for evaluation, but the data that is available for Pulpotec indicated better than clindamycin-modified TAP, mainly concerning furcation radiolucency and results for root resorption [26] (Table 4).

**Table 4:** Technical characteristics of the included studies TAP, triple antibiotic paste; CHX, chlorhexidine; CH, calcium hydroxide; DAP, double antibiotic paste; CS-CHX NPs, chitosan chlorhexidine nanoparticles

Author name	Types of intracanal medicament	Bacterial species targeted	Antibiotic composition and concentration	Duration of intracanal application	Clinical outcomes	Radiographic outcomes	Conclusion
Ahirwar et al. [17]	TAP and *Ocimum sanctum*	Aerobic and anaerobic	Essential oil	3 days	TAP is better than *Ocimum sanctum*	Not applicable	Potential herbal alternative
Chandra and Thosar [18]	3C antibiotic paste vs. TAP	*E. faecalis* and *C. albicans*	Ciprofloxacin, clindamycin, and cefaclor (1:1:1)	Not applicable	Significant bacterial reduction (zone of inhibition measured)	Not applicable	3C paste showed higher efficacy than TAP
Dutta et al. [19]	Calcium hydroxide, TAP, and TAP + CHX	E. faecalis	Ciprofloxacin, metronidazole, and minocycline	1 week	Significant bacterial reduction	Not assessed	TAP + CHX showed superior efficacy
Ghahramani et al. [20]	TAP and Ca(OH)_2_	E. faecalis	TAP mix	7 days	Significant reduction	Not applicable	Effective for resistant bacteria
Gholami et al. [21]	TAP vs. Nano-CH	Aerobic and anaerobic	Ciprofloxacin, metronidazole, and minocycline (20 mg/mL each)	7 days	99.95% aerobic and 99.78% anaerobic reduction (TAP)	Not assessed	TAP demonstrated higher efficacy than Nano-CH
Kargül et al. [22]	Single group: metronidazole	Anaerobic bacteria	Metronidazole 0.1 mL	1 week	75% clinical success	Normal root resorption	Successful for primary dentition
Paikkatt et al. [23]	Ca(OH)_2_, CHX, and metronidazole	Aerobic and facultative anaerobic	1% gel formulations	Not applicable	Ineffective in complete elimination	Not applicable	Not effective in complete elimination
Qamar et al. [24]	Ca(OH)_2_ vs. TAP vs. TAP + CHX	E. faecalis	Ciprofloxacin, metronidazole, and minocycline (1:1:1)	7 days	Significant bacterial reduction	Not assessed	TAP + CHX showed superior efficacy
Reddy et al. [25]	TAP and conventional pulpectomy	NA	3MIX-MP	2 weeks	Excellent success rates	Statistically significant improvement	High success with TAP
Verma et al. [26]	Pulpotec vs. ClinM-TAP	Poly-microbial	Ciprofloxacin, metronidazole, and Clindamycin (43%, 43%, and 14%, respectively)	1 week	88% success (Pulpotec) and 50% success (ClinM-TAP)	60% success (Pulpotec) and 27% success (ClinM-TAP)	Pulpotec showed superior results
Wassel et al. [27]	DAP, CHX, and CS-CHX NPs	*E. faecalis* and *C. albicans*	Various	3 and 7 days	CS-CHX NPs are highly effective	Higher residual effect	CS-CHX NPs recommended

Bias Levels Assessed

**Figure 2:**
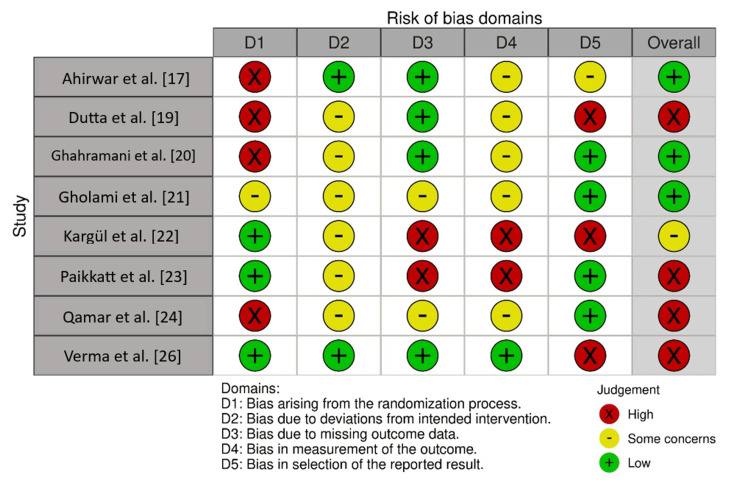
Bias assessment using the RoB 2.0 tool Image credits: The authors of this article.

Domains D1 and D5 showed notable heterogeneity under the RoB 2.0 tool (Figure 2). “High” risk for study design is reported for the studies by Ahirwar et al. [17] and Dutta et al. [19]. In the case of study design, the studies by Kargül et al. [22] and Verma et al. [26] received a “low” risk. For reporting, Paikkatt et al. [23] and Kargül et al. [22] presented “high” risk, whereas others like Gholami et al. [21] and Qamar et al. [24] showed “low” risk. Most studies had “some concerns” in intermediate domains such as D2 (materials and methods) and D4 (experimental controls), indicating a moderate risk of bias in methodological robustness [17-27].

**Figure 3:**
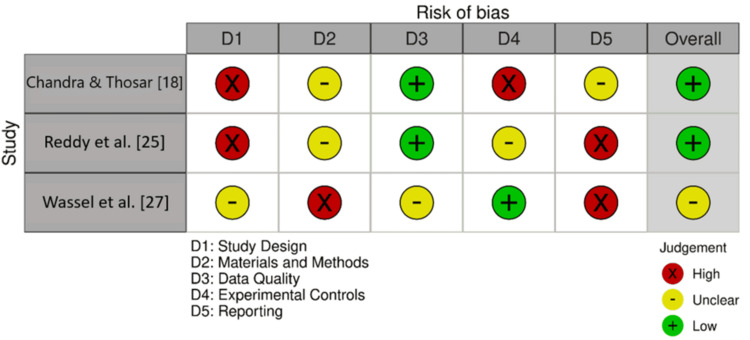
Bias assessment using the QUIN tool Image credits: The authors of this article.

The QUIN tool provided further insights for specific domains (Figure 3). Under the “materials and methods” domain, there were “high” quality studies such as Wassel et al. [27], whereas Chandra and Thosar [18] and Reddy et al. [25] appeared to be of “moderate” quality. Quality of data was again a persisting problem: both Chandra and Thosar [18] and Reddy et al. [25] were rated “low” in this domain. According to Wassel et al. [27], rated “low,” experimental controls were not uniformly applied compared to Chandra and Thosar [18], rated “high.” The reporting quality was high in both Wassel et al. [27] and Reddy et al. [25], suggesting thorough documentation, whereas Chandra and Thosar [18] was rated “moderate.”

Discussion

Dental caries is one of the most common oral infections in children. Not infrequently, it necessitates the removal of most of the structure of the tooth involved due to traditional forms of restorative procedures. Inevitably, this leads to exposure of the pulp or the importance of more invasive restorations. Lesion sterilization and tissue repair (LSTR) is an approach that attempts to directly address infection within the dental caries lesion by promoting natural repair and minimizing invasive restoration procedures [19]. In pediatric patients, the underdevelopment of pain-responsive nerve fibers often delays early diagnosis, and cases present with severe pain that requires invasive interventions such as extractions or pulpectomies [27].

TAP, a constituent of LSTR, is metronidazole, ciprofloxacin, and minocycline, typically in a 1:1:1 ratio. The combination has been modified by substituting minocycline with clindamycin or other appropriate antibiotics, depending on availability and patient-specific requirements. The minimally invasive nature of LSTR avoids extensive instrumentation, which significantly reduces treatment time and enhances patient cooperation, especially in children [28,29].

Ahirwar et al. [17] explored herbal alternatives, finding O. sanctum less effective than TAP but indicating its potential for biocompatibility. This result was much lower than those of Dutta et al. [19] and Qamar et al. [24], which claimed that the effectiveness of TAP plus CHX was much better than that of resistant bacteria. Similar results were observed in Chandra and Thosar [18], in which the 3C antibiotic paste showed greater effectiveness than TAP, partly matching with Gholami et al. [21], where TAP proved better than nano-CH.

Ghahramani et al. [20] highlighted TAP’s efficiency on resistant bacterial species, consistent with Dutta et al. [19] and Qamar et al. [24]; however, in contrast with Paikkatt et al. [23], who observed Ca(OH)₂ and metronidazole formulations as inappropriate for total microbiological elimination.

Verma et al. [26] demonstrated that Pulpotec outperformed clindamycin-modified TAP in radiographic outcomes, showing partial dissimilarity with studies emphasizing TAP’s efficacy. Reddy et al. [25] and Wassel et al. [27] evaluated long-term and nanoparticle-based formulations, respectively, with both highlighting the sustained success of TAP and CS-CHX nanoparticles. These results were in accordance with Qamar et al. [24] and Dutta et al. [19], who had considered the improved antimicrobial activity of TAP-based modifications, though Wassel et al. [27] were the only ones who highlighted extended residual effects.

This technique preserves healthy tooth structures, reduces discomfort, and emphasizes infection elimination and tissue regeneration. LSTR has been refined through various combinations of antibiotics, with ongoing research exploring optimal formulations [30,31]. Evidence supports its ability to improve outcomes in primary teeth by reducing bacterial infections and promoting tissue healing. For instance, clindamycin-based TAP formulations have shown better results than minocycline-based alternatives in certain trials [30,32-34].

Both our review and the study by Malu and Khubchandani [1] have emphasized the need for proper intracanal medicaments to overcome the anatomical challenges of root canals and the broad-spectrum antimicrobial efficacy of TAP. Their evaluation, like our findings, has reaffirmed the role of TAP in effective microbial reduction and infection control, especially in challenging cases.

Achanta et al. [35] highlighted the role of LSTR as a minimally invasive alternative to traditional pulpectomy, similar to our review, which emphasizes TAP and its variations that avoid extensive instrumentation and promote tissue repair. Both studies emphasized the preservation of vital pulp tissues and improved patient cooperation in pediatric endodontics.

Garrocho-Rangel et al. [36] reported high clinical and radiographic success for CTZ paste, similar to the results of TAP-based formulations in our review. Both reviews showed adequate antimicrobial activity and clinical efficacy in the treatment of pulpally involved primary molars, but with differences in the formulations. Agarwal et al. [37] have reported that LSTR had excellent clinical and radiographic success, comparable to that seen in TAP and nanoparticle-based formulations in our review, which underlined the efficacy of LSTR for the management of infected primary teeth.

Malu and Khubchandani [1] focused on the scope of TAP for pulp vitality maintenance, whereas our review was focused on broader issues, including modification in TAP, such as combination with CHX, clindamycin-based formulations, and advanced medicaments based on nanoparticles that provided higher efficiency and longer antimicrobial periods.

Achanta et al. [35] highlighted that LSTR minimized patient pain and made the process of treatment easy; however, this is contrary to our findings, in which some TAP formulations show adverse effects like root resorption. More than that, our systematic review covered more aspects of modifications of TAP formulations and comparative effectiveness, which were not emphasized by Achanta et al. [35]

Garrocho-Rangel et al. [36] reported no significant differences between the clinical, radiographic, and antimicrobial outcomes of CTZ paste and conventional pulpectomy, whereas our review found TAP and its modifications to consistently be superior to the traditional techniques like pulpectomy. Furthermore, biocompatibility concerns of the CTZ paste were reported in their findings, which were less emphasized in our review of TAP formulations.

Agarwal et al. [37] found no statistically significant difference in the success of Vitapex and LSTR. Our review showed that TAP-based therapies surpassed other medicaments for treatment, such as calcium hydroxide and metronidazole formulations. 

Limitations

This review was not without its weaknesses: variability in study designs, small sample sizes, and inconsistent reporting of radiographic outcomes. Protocols for reporting clinical and microbial outcomes were largely standardized, though not uniformly reported. Long-term effects and the possibility of adverse outcomes of new formulations have not yet been explored.

## Conclusions

As a whole, the included studies reveal important information regarding the treatment of infections in primary teeth with tailored intracanal medications. TAP, when either combined with CHX or used as nanoparticles, was always shown to be superior to other antibacterial activities. Novel formulations such as 3C paste and CS-CHX nanoparticles also showed promising results, although they were limited by resistance and discoloration; further clinical validations are required in these areas. In terms of biocompatibility, calcium hydroxide and the less successful herbal alternatives were still useful. The findings emphasize the need for continuous innovation and strict evaluation of intracanal medications to improve therapeutic success and reduce side effects in pediatric endodontics.
